# Anandamide Alters Glycolytic Activity in *Streptococcus mutans*: Metabolomics and Stable Isotope Labeling Study

**DOI:** 10.3390/ijms26178401

**Published:** 2025-08-29

**Authors:** Goldie Wolfson, Doron Steinberg, Alexandra Eliassaf, Anna Morshina, César Jessé Enríquez-Rodríguez, Itzhack Polacheck, Maya Korem, Ori Shalev

**Affiliations:** 1 Institute of Biomedical and Oral Research (IBOR), Faculty of Dental Medicine, The Hebrew University of Jerusalem, Jerusalem 9112102, Israel; goldie.wolfson@mail.huji.ac.il; 2 Department of Clinical Microbiology and Infectious Diseases, Hadassah-Hebrew University Medical Center, Jerusalem 9112001, Israel; itzhackp@ekmd.huji.ac.il (I.P.); mayak@hadassah.org.il (M.K.); 3 The Metabolic Profiling Unit, The Core Research Facility, Faculty of Medicine, The Hebrew University of Jerusalem, Jerusalem 9112102, Israel; 4 Respiratory Medicine Department, Hospital del Mar Research Institute-Hospital del Mar-BRN, 08003 Barcelona, Spain; 5 Medicine and Life Sciences Department, Universitat Pompeu Fabra (UPF), 08003 Barcelona, Spain; 6 Centro de Investigación Biomédica en Red, Área de Enfermedades Respiratorias (CIBERES), Instituto de Salud Carlos III, 28029 Madrid, Spain

**Keywords:** *Streptococcus mutans*, anandamide, carbohydrate metabolism, metabolomics, tracing

## Abstract

*Streptococcus mutans (S. mutans)* is a cariogenic bacterium in the oral cavity that plays a significant role in plaque formation and dental caries. In previous research by our group, we showed that the endocannabinoid anandamide (AEA) has anti-bacterial and anti-biofilm activities against *S. mutans.* Here, we aimed to investigate its effects on *S. mutans* through metabolomics analyses. *S. mutans* was cultivated in the absence or presence of AEA at a sub-minimum inhibitory concentration (MIC), and changes in metabolites and metabolic pathways were assessed through liquid chromatography–mass spectrometry (LC-MS). Treatment of *S. mutans* using AEA at 10 µg/mL significantly disturbed the glycolytic flux in the bacteria, which was indicated by a reduced glucose uptake into the cell, suppression of key glycolytic intermediates, reduced acid production into the media, imbalance of NAD^+^/NADH, and decreased adenosine triphosphate (ATP) production. The disruption of carbohydrate metabolism impacts critical cellular processes, including energy production, redox balance, and biosynthetic pathways, leading to metabolic stress and impaired cellular function. These results highlight the mode of action of AEA as an antimicrobial agent. Altogether, these findings suggest that AEA has potential as a novel antimicrobial agent in the development of therapeutics against *S. mutans*.

## 1. Introduction

The endocannabinoid system, an endogenous system in humans, is composed of (1) the lipid active endogenous ligands *N*-arachidonoylethanolamine (anandamide; AEA) and 2-arachidonoylglycerol (2-AG); (2) biosynthetic enzymes; (3) degradative enzymes; and (4) the CB1 and CB2 cannabinoid receptors (2). The precursors of endocannabinoids (e.g., *N*-acyl-phosphatidylethanolamine (NAPE) and phosphatidylinositol-4,5-bisphosphate (PIP2)) are present in the lipid membranes. Endocannabinoids are produced upon demand, usually after activation of certain G-protein-coupled receptors (GPCRs) and in response to an increase in the intracellular calcium levels [1].

AEA, the first endocannabinoid discovered by R. Mechoulam in 1992 [2], has been extensively studied. Its roles in neural signaling, immune modulation, and physiological regulation are well documented [1,3,4,5]. In vivo studies of AEA have shown its ability to influence cellular processes and metabolic pathways, including appetite control, pain modulation, and cardiovascular and gastrointestinal system functions [1,6,7,8]. Importantly, AEA has not been found to cause mortality in in vivo studies where mice were treated with AEA, even at considerably high concentrations (50 mg/kg) [9]. In contrast to research in humans and mice, AEA’s effects on microorganisms have not been thoroughly investigated. A few studies in recent years have investigated the antimicrobial properties of AEA on several microbes. These studies have observed its activity against pathogens such as methicillin-resistant *Staphylococcus aureus* (MRSA) [1,6,7,8], *Streptococcus salivarius* [10], *Candida albicans* [11], and, recently, *Streptococcus mutans* [12]. These antimicrobial effects may be linked to AEA’s ability to disrupt key metabolic pathways essential for energy production and survival in many microorganisms. However, investigations into AEA’s effects on microbial glycolysis and survival mechanisms remain largely unexplored.

*Streptococcus mutans (S. mutans)* is a cariogenic bacterium in the oral cavity that plays a significant role in plaque formation and dental caries. Dental caries caused by *S. mutans* is a prevalent, widespread disease with a tremendous economic burden [13]. The development of dental caries is initiated by the generation of organic acids from plaque bacteria that lower the pH of the tooth’s surface, which causes demineralization of the tooth enamel [14]. Despite the numerous interspecies interactions and battle for dominance in the plaque biofilms, *S. mutans* is the organism most implicated for dental caries [13]. In reducing or eliminating the disease, much research is focused on targeting *S. mutans* and reducing its presence in dental plaque [13]. Several factors that contribute to its dominance in dental plaque and pathogenicity include (1) its acidogenicity, i.e., ability to ferment sugar to lactic acid rapidly and under considerably low pH conditions which are needed to initiate caries, (2) its aciduricity, i.e., its ability to survive in acidic environments, (3) its strong adherence to tooth surfaces, and (4) its production of extracellular polysaccharides (EPSs) that enhance biofilm formation [13,14,15].

*S. mutans* is able to metabolize a wide variety of carbohydrates through glycolysis—its primary and only energy-generating pathway [15]. Between numerous phosphoenolpyruvate-dependent sugars, phosphotransferase systems (PTSs), and a few ATP-binding cassette (ABC) transporters, various mono-, di-, and oligosaccharides can be internalized, phosphorylated, and processed to fructose-6-phosphate (F6P) and then fermented by glycolysis, where the end results are the production of organic acids—primarily lactic acid [15]. The ability of *S. mutans* to catabolize glucose, fructose, sucrose, and other dietary carbohydrates is a key driver of its pathogenicity, making glycolysis and glucose uptake attractive targets for drug development for therapeutic intervention [16].

Efforts to target *S. mutans* metabolism have highlighted various compounds with potential inhibitory properties. For example, xylitol, fluoride, and *trans*-cinnamaldehyde have been shown to disrupt carbohydrate metabolism by either interfering with glucose uptake or inhibiting specific enzymatic steps in glycolysis [17,18,19,20]. Our previous work demonstrated the anti-bacterial effects of AEA on *S. mutans* [12], but the underlying mechanisms, particularly any distinct effects on glucose uptake and glycolytic flux, remain unclear.

In this study, we examine the metabolic effects of AEA on *S. mutans*, focusing on its dual inhibition of glucose uptake and glycolysis. Using metabolomics and isotopic tracing, we reveal that AEA disrupts bacterial energy production and metabolic homeostasis.

## 2. Results

### 2.1. Metabolic Profiling of S. mutans Treated with AEA

**Figure 1:**
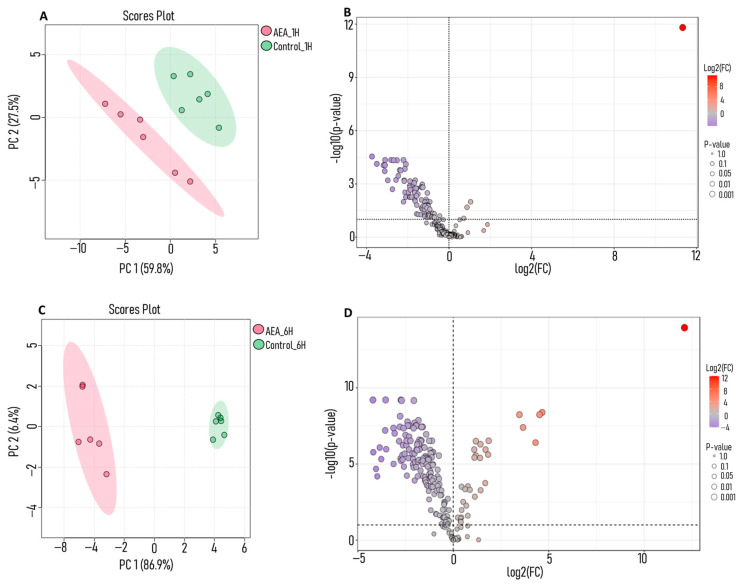
PCA and volcano plot analyses reveal AEA-induced metabolic changes in *S. mutans*. Metabolomic profiling of *S. mutans* treated with AEA. *S. mutans* was untreated (control) or treated with 10 µg/mL for 1 and 6 h. Metabolites were analyzed by LC-MS. PCA score plot of samples at (**A**) 1 h and (**C**) 6 h. Volcano plot showing differentially expressed metabolites in AEA-treated samples at (**B**) 1 h and (**D**) 6 h compared to controls. Data were analyzed and visualized using MetaboAnalyst software V.6.0. *n* = 6.

Metabolic profiling was conducted on *S. mutans* treated with AEA (10 µg/mL, below the minimum inhibitory concentration (MIC); Supplementary Materials Figure S1). Samples were taken at two time points (1 and 6 h) and analyzed using liquid chromatography–mass spectrometry (LC-MS)-based metabolomics to analyze alterations in metabolic pathways. Overall, 208 metabolites were identified. Table S1 lists all 208 identified metabolites, including their classifications and pathway associations. The principal component analysis (PCA) score plot (Figure 1A,C) illustrates the unbiased groupings between the control samples (green) and the AEA-treated samples at both time points. At both 1 h (Figure 1A) and 6 h (Figure 1C), there is a clear separation between the control and treated groups with a complete lack of overlap in the ellipses of each group, indicating a strong difference in the treatment effect.

On a volcano plot, of the 208 metabolites identified in the study, 155 were downregulated in AEA-treated samples after 1 h, with 96 calculated to be statistically significant (Figure 1B). After 6 h, 164 were downregulated, with 151 being identified as statistically significant (Figure 1D) compared to controls. Furthermore, analysis of the downregulated metabolites revealed that a subset of statistically significant changes at both the 1 and 6 h time points was associated with glycolysis. This suggests a targeted disruption of glucose metabolism after just 1 h of incubation, which is still maintained after 6 h. *p*-values for the metabolites are listed in Table S2 for 1 h and Table S3 for 6 h.

### 2.2. Key Glycolytic Intermediates Are Suppressed

**Figure 2:**
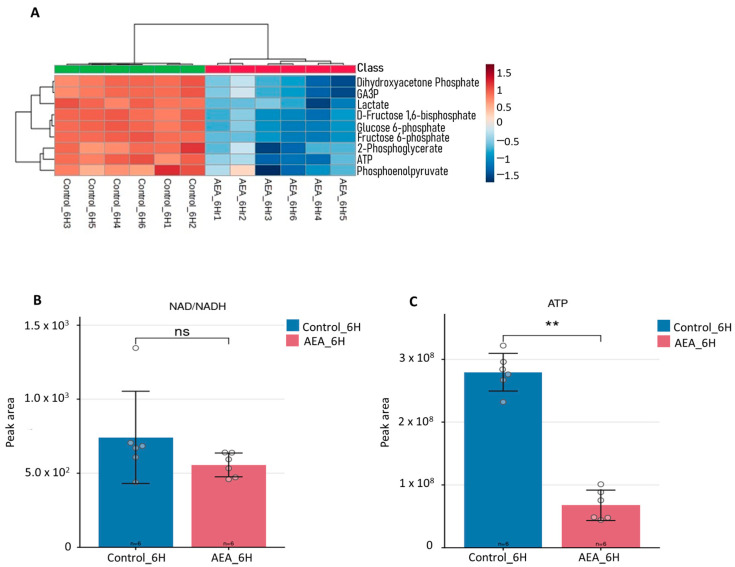
ATP production is suppressed, and NAD^+^/NADH imbalance reflects redox disruption. (**A**) Heatmap showing glycolytic intermediates after 6 h incubation with 10 µg/mL AEA, displaying glycolytic intermediates glyceraldehyde-3-phosphate (GA3P), dihydroxyacetone phosphate, lactate, D-fructose 1,6-biphosphate (F1,6BP), F6P, 2-PG, ATP, and phosphoenolpyruvate. Samples were analyzed and graphs were generated using MetaboAnalyst software V.6.0. *n* = 6. (**B**) NAD^+^/NADH ratio and (**C**) ATP production in AEA-treated samples after 6 h incubation compared to control. Samples were analyzed and graphs were generated using Metabolite Autoplotter V.2.6. *n *= 6. ** *p* < 0.01; n.s. not significant.

To validate the findings from the untargeted analysis in Figure 1, a more focused analysis of glycolytic intermediates was conducted. Heatmap analysis indicated that key intermediates in the glycolysis cycle were significantly reduced in treated cells compared to controls (Figure 2A). Levels of glucose-6-phosphate (G6P) and fructose-6-phosphate (F6P), which represent early glycolytic steps, were markedly decreased, suggesting a limitation in glucose uptake or entry into the glycolysis cycle (Figure 2A). Similarly, fructose-1,6-bisphosphate (F1,6BP), a key intermediate at the committed step of glycolysis, was significantly lower in treated cells (Figure 2A). These reductions indicate that AEA inhibits early glycolytic activity, thereby restricting carbon flow through the pathway.

Further downstream, intermediates of mid- and late glycolysis, including 2-phosphoglycerate (2-PG) and phosphoenolpyruvate (PEP), were also significantly reduced in treated cells (Figure 2A). The suppression of these intermediates highlights a metabolic bottleneck, further limiting the conversion of glucose to pyruvate. Consistent with this observation, pyruvate, an important end product of the glycolysis pathway, was significantly reduced under treatment conditions relative to controls (Figure 2A). The coordinated downregulation of glycolytic intermediates suggests that AEA treatment broadly suppresses glycolytic flux, impairing glucose-derived energy production.

#### 2.2.1. NAD^+^/NADH Imbalance Reflects Redox Disruption

The NAD+/NADH ratio, a key indicator of cellular redox balance, was reduced in AEA-treated cells compared to controls (Figure 2B), indicating a disruption in redox homeostasis. This reduction impairs NAD+ regeneration, which is essential for glycolysis, where NAD+ acts as an electron acceptor during the oxidation of glyceraldehyde-3-phosphate. Consequently, the decreased NAD+/NADH ratio likely diminishes glycolytic activity and energy production. In *S. mutans*, which lacks a complete electron transport chain, maintaining the NAD+/NADH balance is critical for sustaining glycolytic flux and overall metabolic function. The ratio imbalance was associated with NAD^+^ accumulation in both control and AEA-treated samples, with a slower accumulation observed in the AEA-treated group compared to controls, rather than a depletion of NADH. The observed decrease in this ratio, therefore, contributes to metabolic disruption and impaired cellular processes under AEA treatment.

#### 2.2.2. Compromised Adenosine Triphosphate (ATP) Production

ATP levels, a direct indicator of energy metabolism, were statistically significantly lower after 6 h incubation with AEA, showing a 75% reduction compared to controls (Figure 2C), which is consistent with the observed impairment of glycolytic activity. Glycolysis is a primary source of ATP production, particularly under glycolytic conditions. These results demonstrate that AEA-induced glycolytic suppression directly compromises energy availability, likely impacting cell function and survival.

### 2.3. Pathway Enrichment and Impact Analysis

**Figure 3:**
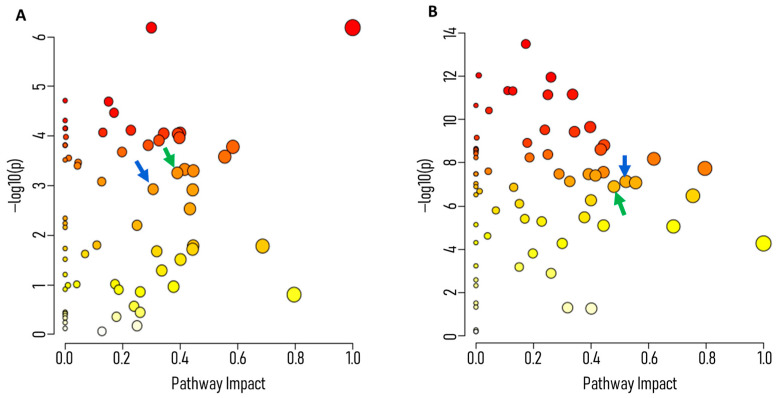
Pathway enrichment and impact analysis of *S. mutans* treated with AEA. *S. mutans* was untreated (control) or treated with 10 µg/mL AEA. Metabolites were analyzed by LC-MS. Pathway Enrichment Analysis results are shown after (**A**) 1 h and (**B**) 6 h of incubation. Green arrows indicate the PPP, and blue arrows denote the glycolysis pathway. Data were analyzed and visualized using MetaboAnalyst Software V.6.0. *n* = 6.

Untargeted metabolomics data were analyzed to assess potential pathway disruptions induced by AEA. Pathway impact scores for glycolysis revealed progressive inhibition over time. At 1 h, glycolysis exhibited moderate disruption, with an impact score of 0.39029 and an FDR-adjusted *p*-value of 0.001271 (Figure 3A). By 6 h, the impact score increased to 0.4799, accompanied by a highly significant FDR-adjusted *p*-value of 2.24 × 10^−7^ (Figure 3B). This escalation could indicate cumulative metabolic stress caused by sustained AEA treatment.

Pathway visualization further demonstrated significant perturbations in associated pathways, including the pentose phosphate pathway (PPP) and pyruvate metabolism, highlighting the broader impact of AEA on central carbon metabolism. The pathway impact values are listed in Table S4 for 1 h samples and Table S5 for 6 h samples. These findings provide strong justification for further targeted and tracing analyses to delineate the specific mechanisms underlying these disruptions.

### 2.4. Tracing of Intracellular Metabolites

To further investigate the impact of AEA on glycolytic metabolism, we performed a stable isotope tracing experiment using uniformly labeled D-Glucose (U-^13^C_6_). This approach enabled us to monitor the incorporation of labeled carbon atoms into glycolytic intermediates and assess pathway activity under control and treated conditions.

**Figure 4:**
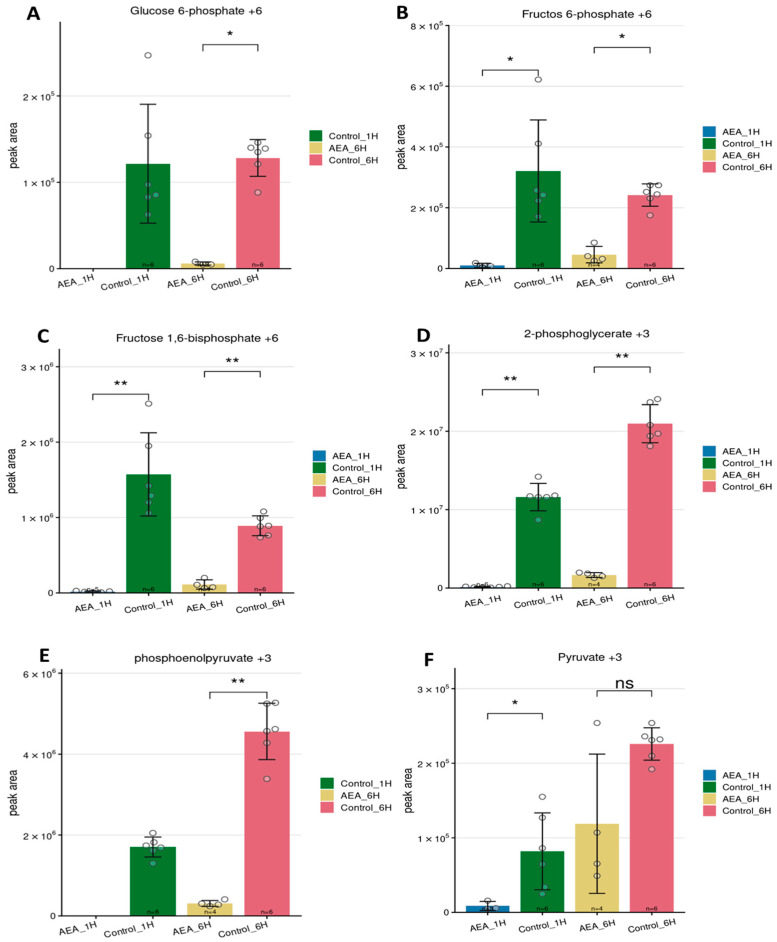
Tracing analysis of glycolytic intermediates. *S. mutans* untreated and treated with 10 µg/mL AEA for 1 and 6 h, grown in the presence of ^13^C-glucose, and analyzed by LC-MS-based metabolomics. Glycolytic intermediates (**A**) G6P, (**B**) F6P, (**C**) F1,6BP, (**D**) 2-PG, (**E**) PEP, and (**F**) pyruvate were analyzed, and graphs were generated using Metabolite AutoPlotter V.2.6. Statistical significance was calculated using the Mann–Whitney test. *n *= 6 for all conditions except AEA 6 h, where *n *= 4. * *p* < 0.05 and ** *p* < 0.01; n.s. not significant.

At the 1 h time point, significant ^13^C enrichment was detected in early glycolytic intermediates, including glucose-6-phosphate (G6P +6), fructose-6-phosphate (F6P +6), and fructose-1,6-bisphosphate (F1,6BP +6). These metabolites were markedly lower in AEA-treated cells compared to controls (Figure 4A–C), indicating reduced glucose uptake or impaired enzymatic conversion at the upper glycolysis level. This early inhibition was also reflected in mid- and late-stage intermediates: 2-phosphoglycerate (2-PG +3) and phosphoenolpyruvate (PEP +3) showed reduced labeling in the AEA group (Figure 4D,E), suggesting consistent pathway suppression downstream.

By the 6 h time point, accumulation of labeled intermediates in the control group continued to rise, while AEA-treated samples remained low across nearly all measured metabolites. Notably, the gap between treated and control groups widened at 6 h, especially for intermediates such as PEP and 2-PG, pointing to sustained inhibition of glycolytic flow. For pyruvate (+3), although the difference between control and treated was less pronounced, enrichment levels were still lower under AEA exposure (Figure 4F). Together, these results reveal a persistent and time-dependent reduction in ^13^C enrichment across glycolytic intermediates in AEA-treated *S. mutans*, reflecting a suppressed metabolic response relative to the control. Complete lists of labeled intracellular and extracellular metabolites are provided in Tables S6 and S7, respectively.

### 2.5. Tracing Analysis of Glucose and Lactate in the Growth Media

**Figure 5:**
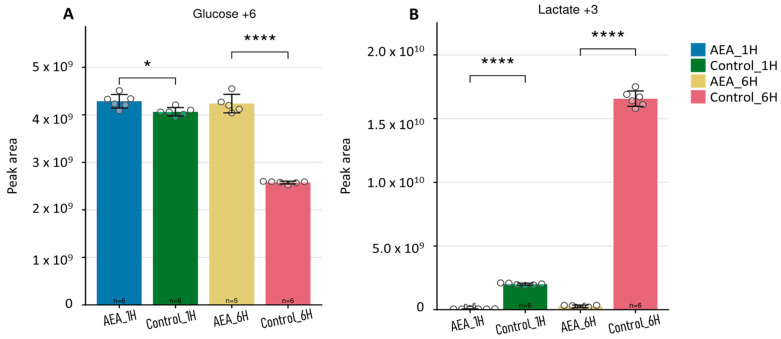
Metabolomics of extracellular glycolytic intermediates. Glycolytic intermediates (**A**) ^13^C_6_-glucose and (**B**) ^13^C_3_-lactate were analyzed, and graphs were generated using Metabolite AutoPlotter V.2.6. *n *= 6 for all conditions except AEA 6 h, where *n *= 5. * *p* < 0.05 and **** *p* < 0.0001.

Analysis of extracellular ^13^C_6_-glucose levels in the growth media of the bacteria revealed statistically significant differences between control and treated groups over the incubation period (Figure 5). The results observed from ^13^C_6_-glucose (Figure 5A) show a clear decrease in the ^13^C_6_-glucose by the 6 h time point compared to 1 h, implying glucose has successfully been taken up into the cell. However, in the AEA-treated group, the levels of ^13^C_6_-glucose after 6 h remain close to the levels at 1 h in a highly significant manner (Figure 5A).

Furthermore, lactate, the end product of glycolysis, is expected to be expelled from the cell into the media. In the control groups, there is a marked increase in ^13^C_3_-lactate found in the growth media by the 6 h time point (Figure 5B). This reflects active glycolysis metabolism in untreated cells, with a significant conversion of pyruvate to lactate. In contrast, the AEA-treated groups displayed a markedly reduced ^13^C_3_-lactate production concomitant with the lack of glucose uptake, further supporting the notion that there is an inhibition of glycolysis.

### 2.6. Flux Analysis of Intracellular Glycolytic Intermediates in the Growth Media

**Figure 6:**
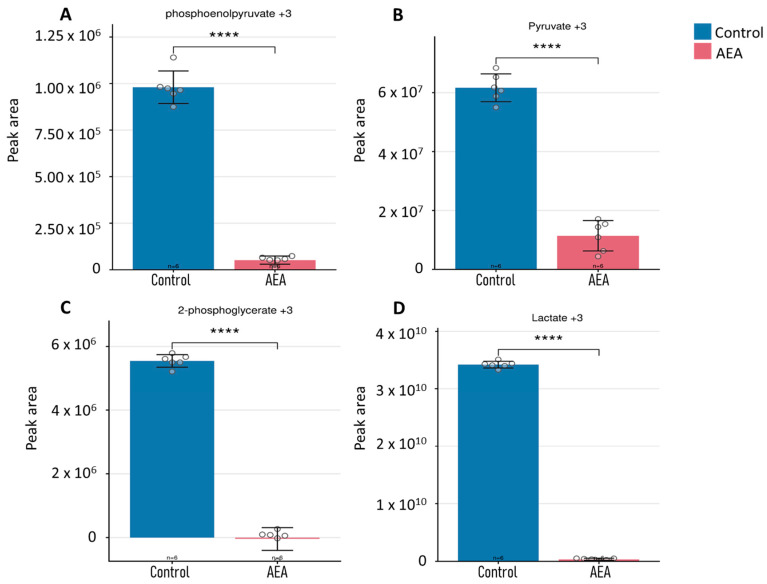
Metabolomics and flux analysis of intracellular glycolytic intermediates. Intracellular glycolytic intermediates, including (**A**) ^13^C_3_-phosphoenolpyruvate, (**B**) ^13^C_3_-pyruvate, (**C**) ^13^C_3_-2-PG, and (**D**) ^13^C_3_-lactate, were analyzed, and graphs were generated using Metabolite AutoPlotter V.2.6. *n* = 6. **** *p* < 0.0001.

To complement the intracellular labeling data, we analyzed the isotopic enrichment of glycolytic end products secreted into the extracellular medium. These measurements reflect the rate at which metabolites are produced and secreted through the glycolysis pathway over time (1 to 6 h). This provided an additional perspective on pathway activity and metabolite export in response to AEA treatment. For PEP, enrichment increased more substantially in control cells compared to treated cells, suggesting reduced glycolytic progression in the presence of AEA (Figure 6A). Similarly, although pyruvate enrichment showed some variability, it was consistently lower in treated cells, supporting an inhibitory effect of AEA on glycolytic processing (Figure 6B). Enrichment of 2-PG was also reduced in treated cells relative to controls, indicating suppression across glycolytic intermediates (Figure 6C). The most pronounced reduction was observed for lactate, where enrichment levels were significantly lower in AEA-treated cells, reflecting diminished pyruvate-to-lactate conversion under treatment (Figure 6D). These findings collectively indicate that AEA treatment leads to a broad reduction in glycolytic activity, limiting glucose-derived energy production in *S. mutans.*

These findings from the growth media analysis corroborate the intracellular data, indicating that AEA treatment impairs glucose transport or utilization, contributing to the observed glycolytic inhibition.

### 2.7. AEA Treatment Reduces Growth Rate, Aciduricity, and Acidogenicity

**Figure 7:**
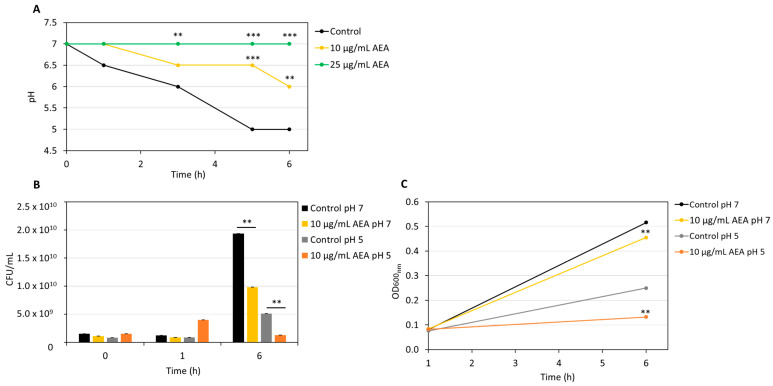
AEA reduces aciduricity and acidogenicity in *S. mutans.* (**A**) The pH of the *S. mutans* culture medium measured at 0, 1, and 6 h incubation with AEA (0, 12.5, and 25 µg/mL). (**B**) CFUs per mL of bacteria treated with 0 and 10 µg/mL AEA in pH 7 and pH 5.2 after 0, 1, and 6 h of incubation. (**C**) The OD of these samples measured at the same time. *n* = 3; ** *p* < 0.01, *** *p* < 0.001.

The effect of AEA on *S. mutans* growth was assessed by measuring the optical density (OD) of planktonic cultures grown in the presence of 12.5 and 25 µg/mL AEA for over a period of 24 h. AEA treatment slowed the growth in a dose-dependent manner, with greater inhibition observed at 25 µg/mL (Figure S1). Given that acidogenicity is a key virulence factor of *S. mutans* and a direct consequence of glycolysis, we also investigated the effect of 10 µg/mL AEA on the pH of the media. While the pH of control cultures reached 5 after 6 h, reflecting significant acid production, the pH of cultures treated with 10 µg/mL AEA only reached 6.5. In cultures treated with 25 µg/mL AEA, the pH remained at 7 (Figure 7A), indicating a substantial reduction in acid production in the 10 µg/mL AEA-treated samples.

Aciduric is another important virulence factor for *S. mutans.* Therefore, we investigated the effect of AEA on acid tolerance by comparing the number of colony-forming units (CFUs) of *S. mutans* grown in the absence (control) and presence of 10 µg/mL AEA at pH 7 and pH 5.2 (Figure 7B). After 1 h of incubation with AEA, CFU counts were similar between treated and control groups at both pH levels.

However, after 6 h, AEA-treated samples at pH 7 showed a 2-fold reduction in CFUs compared to their respective controls, as well as a 4-fold reduction at pH 5.2 compared to their respective controls (Figure 7B). The OD of these samples was measured simultaneously, revealing a concomitant reduction in OD in the presence of AEA at both pH 7 and pH 5 compared to their respective controls (Figure 7C).

### 2.8. The Effect of AEA on Carbohydrate Uptake and Glycolysis

**Figure 8:**
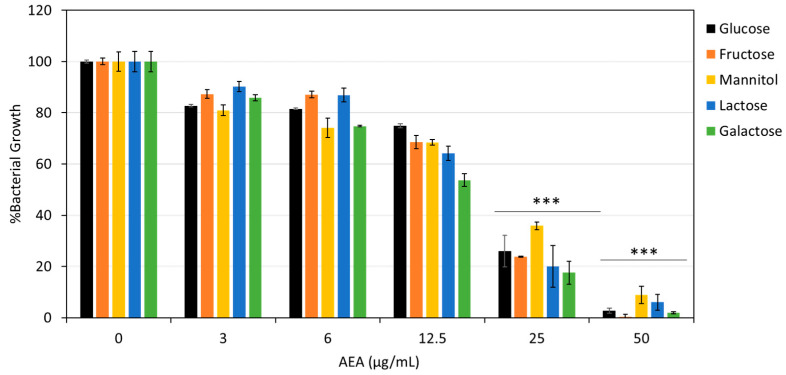
AEA inhibits various carbohydrate metabolisms. The viability of *S. mutans* after 24 h incubation with AEA (0–50 µg/mL) with the external addition of either glucose, fructose, mannitol, lactose, or galactose to a final concentration of 1% measured at OD_600nm_. *n* = 3; *** *p* < 0.001.

To determine whether AEA’s effect on *S. mutans* was specific to glucose uptake or involved a broader mechanism, we performed a carbohydrate rescue experiment. *S. mutans* was grown in tryptic soy broth (TSB) supplemented with 1% final concentration of various carbohydrates. Several different carbohydrates were used in this experiment. After 24 h of incubation with varying concentrations of AEA (0–50 µg/mL), we observed that the MIC of AEA remained between 25 and 50 µg/mL regardless of the presence of different carbohydrates such as fructose, mannitol, lactose, and galactose (Figure 8). These results indicate that AEA’s inhibitory effect is not solely due to impaired glucose uptake.

## 3. Discussion

The oral microflora is remarkably complex, harboring a vast diversity of microorganisms [21]. Bacteria are the most abundant microorganisms, with Streptococci species, particularly *S. mutans*, dominating the cariogenicity of the oral cavity [21]. *S. mutans* plays a critical role in dental plaque biofilm formation and is the primary etiologic agent of dental caries [21]. Its virulence is largely attributed to its aciduricity and acidogenicity [14,21]. Despite the availability of antibiotics and antiseptics targeting *S. mutans* and other cariogenic bacteria, effective treatments that fully eliminate their virulence remain elusive. Consequently, there has been a continuous search for novel therapeutic strategies against cariogenic species, focusing on antimicrobial, anti-biofilm, and, lately, antimetabolic approaches, particularly those that target acid production.

Previous research by our group demonstrated that AEA impacts *S. mutans* by affecting cell viability, biofilm formation via extracellular polysaccharide (EPS) production, the cell membrane, general metabolomics, and changes in cell morphology [12]. Importantly, the concentrations of AEA needed for the anti-bacterial effects were below the cytotoxic concentration for normal Vero epithelial cells [12]. Based on these previous findings, in the present study, we explored AEA’s mode of action via its effect on cell metabolism, specifically in the presence of glucose. Glucose is an essential nutrient in bacterial fermentation. It is a crucial energy source that results in the production of organic acids, which cause tooth demineralization and cavitation [14].

In our previous study, we performed experiments in brain heart infusion (BHI) media^8^, whereas in this study, we chose to use TSB media so we could control the levels of glucose the bacteria were exposed to. We observed the only notable difference to be in the MIC, which was 12.5 µg/mL in BHI to 25 µg/mL in TSB. This variation is expected, as previous studies have demonstrated that MIC values can be significantly influenced by growth medium composition, including factors such as nutrient content, buffering capacity, and even assay plate material, in both *S. mutans* and other bacterial species [22,23,24,25].

In the present study, we used *S. mutans* as a cariogenic bacterial model and demonstrated that AEA disrupts glycolysis in this bacterium. We observed that AEA reduces glycolytic activity flux and consequently diminishes acid production, thereby attenuating its cariogenic virulence. 

A number of studies have investigated the effects of various compounds on *S. mutans*, revealing notable alterations in bacterial metabolism and glycolysis. For instance, fluoride treatment is known to inhibit bacterial growth by partially targeting the glycolytic enzyme enolase, thereby reducing glucose metabolism [20]. Xylitol, a sugar alcohol, has also been shown to inhibit *S. mutans* growth. Once taken up and phosphorylated to xylitol-5-phosphate (X5P), this compound cannot be further metabolized, leading to its intracellular accumulation. The buildup of X5P disrupts glycolytic intermediates and ultimately suppresses sugar metabolism [20]. To further study and elucidate the mechanism by which AEA affects *S. mutans*, our group designed and conducted a novel metabolomics experiment.

After performing untargeted metabolomic analysis, notably, many of the significantly downregulated metabolites were glycolytic intermediates (Figure 1 and Figure 2A). Furthermore, pathway analysis (Figure 3) indicated that the glycolysis pathway had been altered in a significant way compared to control samples. To further understand and investigate the importance of this, we chose to perform a tracing analysis experiment with labeled glucose to better observe the effects of AEA on the central carbon metabolism.

Tracing experiments revealed a significant reduction in the incorporation of ^13^C_6_-glucose into both early and late glycolytic intermediates, demonstrating the inhibitory effects of AEA across the glycolytic pathway.

The isotopic tracing enrichment flux analysis further supports these findings, showing a marked decrease in the incorporation of labeled glucose in intracellular intermediates and the reduction in labeled extracellular pyruvate and lactate end products. The reduction in lactate is particularly important, since it plays a central role in the cariogenic potential of *S. mutans*, contributing to acid production and biofilm formation. In the combined treatment of fluoride and xylitol, a reduction in lactate production in *S. mutans*, accompanied by a reduction in biofilm formation and overall virulence of the bacteria, was also observed [20]. When *S. mutans* is treated with nicotinamide, again, the same results are observed, in addition to a reduction in acid production, a concomitant reduction in biofilm formation, and EPS production [19]. By limiting lactate production, AEA not only restricts glycolysis but also impedes the cariogenic virulence properties of *S. mutans*.

The impact of this disruption is further reflected in the decline in ATP levels. ATP is crucial for energy metabolism in *S. mutans*, as it is utilized for numerous biological pathways and processes, including fermenting carbohydrates into lactic acid, cell wall synthesis, membrane transport systems, protein synthesis, DNA synthesis, pH homeostasis, biofilm formation, and DNA repair [14,26,27]. The declining levels of ATP caused by AEA in *S. mutans* can be observed by the reduced production and export of acid (lactate) out of the cell (Figure 5B and Figure 7A), the damages to the membrane transport and biofilm formation that was noted previously [12], and the dysregulation in pH homeostasis that can be noted by the reduced tolerance to acidic conditions (Figure 7B,C).

*S. mutans* treated with bedaquiline showed inhibited H^+^-ATPase activity, which was accompanied by reduced bacterial growth, biofilm formation, EPS secretion, and lactic acid production [28]. Similarly, treatment with piceatannol, curcumin, and demethoxycurcumin also resulted in reduced F-type ATPase activity, which is essential for *S. mutans* survival under acidic conditions [29]. These findings are consistent with observations from the current study on AEA-treated *S. mutans.* Notably, the effects of bedaquiline, piceatannol, curcumin, and demethoxycurcumin were most pronounced under acidic conditions (pH 5) and showed minimal impact at neutral pH (pH 7) [28,29]. While it is known that F-ATPase expression is upregulated under acidic conditions, the lack of any significant effect at neutral pH underscores the need to further elucidate the precise mechanisms of action of these compounds [29]. Therefore, given that *S. mutans* primarily relies on glycolysis for ATP generation, targeting ATP and the consequent reduction in energy availability, compromises its aciduricity, ability to sustain growth, pH homeostasis, and maintenance of biological pathways, and thus, it becomes less virulent in the presence of AEA even in a more neutral pH.

*S. mutans* has complex stress response pathways that allow it to survive the rapidly changing environment of the oral cavity, surpass competing microbial pathogens, and thrive in the dental plaque biofilm [13], which can mainly be attributed to its robust response to both acid and oxidative stress [30]. These responses are mostly attributed to NADH oxidase, which is thought to be critical for the regeneration of NAD^+^ for use in glycolysis and for the reduction of oxygen, which in turn prevents the formation of damaging reactive oxygen species [30]. The balance between oxidized NAD^+^ and reduced NADH is central to cell homeostasis. Thus, the observed shift in the NAD^+^/NADH ratio after AEA treatment suggests an imbalance in redox homeostasis, which can support our observations that AEA disrupts carbon metabolism and bacterial survival and reduces its stress response and resistance.

The reduction in the NAD^+^/NADH ratio, alongside suppressed ATP production, highlights a dual impairment in energy metabolism and redox homeostasis. Together, these findings indicate that treatment disrupts glucose utilization and energy production while exacerbating redox imbalance, further compounding metabolic stress. These effects collectively compromise cell viability and metabolic flexibility, exacerbating the cellular response to treatment.

A signature characteristic of *S. mutans* is its ability to metabolize a large variety of carbohydrates [15]. The PTS is a major carbohydrate transport system in streptococci, especially under carbohydrate-limiting conditions [31]. There are two general proteins that make up the PTS: the enzyme I (EI) and the heat-stable phosphocarrier protein (HPr). Additionally, the presence of enzyme II complexes (EII) allows for the phosphorylation and internalization of many different sugars into the bacteria [31]. The *S. mutans* UA159 strain specifically encodes 14 phosphoenolpyruvate-dependent sugar: PTSs with specificities for various mono- and disaccharides and additional ABC transporters for oligosaccharides [15].

This evolution of multiple transporters and regulatory capacities ensures the bacteria will be able to catabolize a wide range of carbohydrates and tolerate major fluctuations in nutrient availability and pH, which is crucial for their survival and cariogenicity [15,31]. We observed that AEA impacted more than just glucose uptake since the MIC of AEA remained between 25 and 50 µg/mL regardless of the presence of fructose, mannitol, lactose, and galactose (Figure 8). These results indicate that AEA’s inhibitory effect hinders the uptake and utilization of numerous carbohydrate sources, most likely affecting the EII enzyme complex in the bacteria, since this is the primary mode of uptake of various carbohydrates into the cells. It would be worthwhile to further investigate the binding site of AEA with sugar-specific permeases such as the EII complexes.

Notably, when comparing AEA to chlorhexidine (0.0075–2.0 mM), a widely accepted treatment used against cariogenic oral bacteria, there is a similar inhibition of bacterial growth and lactic acid production and a reduction in glycolytic intermediates and the rate of glycolysis after chlorhexidine treatment [32]. Further investigation into how chlorhexidine affects glycolysis revealed that it primarily targets the cell membrane of *S. mutans*, causing glycolytic intermediates to leak out of the cell rather than directly inhibiting the glycolytic pathway itself, as was originally thought [32]. In contrast, this mechanism was ruled out for AEA treatment, as only minimal amounts of labeled intermediates were detected in the growth medium (Figure 6).

Additionally, in the case of *trans*-cinnamaldehyde and triclosan, which both inhibit the growth rate and biofilm formation of *S. mutans*, they were both found to interfere with the glycolysis pathway of *S. mutans* [17,33] through various mechanisms. Investigations on the former suggest it suppresses glucose and sucrose consumption and interacts with pyruvate dehydrogenase (PDH), downstream of glycolysis, causing an anti-bacterial effect on *S. mutans* [17]. The latter has been observed to affect the bacteria through inhibition of glycolytic enzymes such as pyruvate kinase, lactate dehydrogenase, and aldolase [33].

The observations of this study demonstrate that AEA treatment significantly disrupts glycolysis in *S. mutans*, leading to reduced glucose uptake, lower intracellular glycolytic metabolite levels, and a decline in extracellular acid production. The alignment between increased extracellular glucose and the depletion of key glycolytic intermediates suggests that AEA impairs glucose transport or early glycolytic processing, thereby limiting the entry of carbon into the pathway. One notable novelty is that, unlike the other agents discussed here, which act directly on glycolytic enzymes, the cell membrane, or the ATPase activity, AEAs’ effect is primarily seen at the level of carbohydrate uptake. This inhibition propagates downstream, reducing the availability of pyruvate and lactate, the major fermentation end products that drive *S. mutans* acidogenicity. The substantial decrease in lactate production impedes *S. mutans*’ cariogenic potential by diminishing its glycolytic capacity flux, thus reducing its acid production, and thereby weakening the ability of *S. mutans* to generate the acidic microenvironment necessary for enamel demineralization and biofilm stability.

## 4. Materials and Methods

### 4.1. Materials

AEA (>98.0% purity) was purchased from Cayman Chemical (Ann Arbor, MI, USA) and dissolved in absolute ethanol at a concentration of 10 mg/mL. Respective dilutions of ethanol (0.0156–0.5%) as well as untreated bacteria were used as controls in this study.

### 4.2. Bacterial Growth Media

TSB media was prepared in the following manner: Tryptone (8.5 g/L) (Acumedia, Lansing, MI, USA), Peptone (1.5 g/L) (HiMedia Laboratories, Mumbai, India), NaCl (2.5 g/L), and K_2_HPO_4_·3H_2_O (2.3 g/L) were added, mixed, and autoclaved together. Glucose was prepared separately, filter-sterilized, and added to a final concentration of 1% to the TSB media prior to use (TSBG).

In the carbohydrate rescue experiment, the TSB was used with the addition of fructose, mannitol, galactose, or lactose. All carbohydrates were prepared in the same manner as the glucose and added to a final concentration of 1% to the TSB.

For the tracing analysis experiment, D-Glucose (U-^13^C_6_, 99%) (CLM-1396-1) was purchased from Cambridge Isotope Laboratories, Inc. (Andover, MA, USA), and was diluted directly into TSB and sterile-filtered before use at a final concentration of 1%.

### 4.3. Bacterial Growth

*S. mutans* UA159 was grown overnight at 37 °C in 95% air/5% CO_2_ in TSBG until an OD of approximately 1.2–1.3 was reached [12]. For planktonic growth, the overnight culture was diluted to an OD_600nm_ of 0.1 in TSBG, and 200 µL of the bacterial culture was seeded in each well of a tissue-grade flat-bottom 96-well microplate (Corning, Glendale, AZ, USA) in the absence or presence of increasing concentrations of AEA (1.25–50 µg/mL) or respective ethanol concentrations (0.0156–0.5%) and incubated at 37 °C in 95% air/5% CO_2_ for 24 h.

### 4.4. LC-MS-Based Metabolomics

#### 4.4.1. Sample Preparation

This protocol was adapted from Mielko et al. [34]. Overnight bacterial suspensions of OD_600nm_ of 0.1 in the TSBG medium were centrifuged at 15,000× *g* for 10 min and washed twice in TSB media in order to remove any unlabeled glucose from the media. After this, an OD_600nm_ of 0.1 of *S. mutans* was resuspended in the TSBG medium for metabolomic profiling or TSB + D-Glucose (U-^13^C_6_, 99%) (CLM-1396-1) (final concentration of 1%) for metabolomic tracing. The bacteria were then either left untreated or treated with a sub-MIC of AEA (10 µg/mL) and incubated at 37 °C, in air/5% CO_2_, for 1 and 6 h.

After incubation, from each sample, an OD_600nm_ of 0.1 was taken in order to standardize the experiment, which was centrifuged at 15,000× *g* for 10 min at 4 °C and washed in order to separate the supernatant from the cell pellets. The cell pellets and supernatant were collected separately in Eppendorf tubes, and the cell pellet was washed twice in cold phosphate-buffered saline (PBS), which was then removed. The cell pellet was carefully aspirated to ensure a clean pellet, and the pellet and media samples were transferred to glass high-performance liquid chromatography (HPLC) vials and stored at −80 °C prior to LC-MS analysis. Untreated samples served as controls.

For intracellular metabolite extraction, the pellets were treated with 100 µL of an ice-cold extraction mixture composed of methanol, acetonitrile, and water in a 5:3:2 ratio. The samples then underwent three freeze–thaw cycles to enhance metabolite extraction. For media extraction, 20 µL of medium was mixed with 180 µL of the same extraction mixture. Following extraction, the samples were centrifuged at 20,000× *g* for 20 min at 4 °C. The resulting supernatant was carefully collected and transferred to HPLC vials for analysis. Quality control (QC) samples were prepared by pooling equal aliquots from each sample group. Additionally, for MS/MS analysis, each group was pooled to create an “ID sample” to facilitate metabolite identification.

#### 4.4.2. LC-MS Data Acquisition

LC-MS metabolomics analysis was performed as described previously [35]. Briefly, a Dionex Ultimate 3000 high-performance liquid chromatography (UPLC) system (Thermo Fisher Scientific, Germering, Germany) coupled to an Orbitrap Q-Exactive plus Mass spectrometer (Thermo Fisher Scientific, Bermen, Germany), with a resolution of 70,000 at a 200 mass/charge ratio (*m*/*z*), electrospray ionization in the HESI source, and polarity switching mode to enable both positive and negative ions across a mass range of 70 to 1000 *m*/*z*, was used. The UPLC setup included a ZIC-pHILIC column (SeQuant, Merck, Darmstadt, Germany; 150 mm × 2.1 mm, 5 μm) with a Sure-Guard filter (SS frit 0.5 μm) (Thermo Fisher Scientific, Waltham, MA, USA). Of the sample extracts, 5 μL was injected, and the compounds were separated with a mobile phase gradient of 15 min, starting at 20% aqueous (20 mM ammonium carbonate adjusted to pH 9.2 with 0.1% of 25% ammonium hydroxide) and 80% organic (acetonitrile) and terminated with 20% acetonitrile. The flow rate and column temperature were maintained at 0.2 mL/min and 45 °C, respectively, for a total run time of 26 min. All metabolites were detected using mass accuracy below 5 ppm. Thermo Xcalibur (Thermo Fisher Scientific, Waltham, MA, USA) was used for data acquisition. Metabolomics data was normalized to optical density.

#### 4.4.3. Metabolomics Data Analysis

Data processing and analysis were performed using Compound Discoverer (version 3.3.2.31) (Thermo Fisher Scientific, Bermen, Germany) with an in-house library. Tracing data analysis was conducted using Trace Finder (version 5.2) (Thermo Fisher Scientific, Bermen, Germany).

#### 4.4.4. Metabolomics Statistical Analysis

All statistical analyses for the metabolomics experiments were performed using appropriate bioinformatics tools and statistical software. Untargeted metabolomics data were processed and analyzed using MetaboAnalyst software (version 6.0) (Xia Lab, McGill University, Montréal, QC, Canada), which was used for data normalization, feature selection, and visualization. Volcano plots were generated in MetaboAnalyst software (Xia Lab)**,** applying a two-sample *t*-test or Wilcoxon rank-sum test for statistical comparisons, which is consistent with the software’s differential analysis module.

For targeted metabolite comparisons, statistical analysis was performed using Metabolite AutoPlotter (version2.6) (West Coast Metabolomics Center, University of California, Davis, CA, USA)**,** employing a two-tailed Mann–Whitney U nonparametric test to assess differences between experimental groups.

Pathway impact analysis was conducted using MetaboAnalyst software (Xia Lab)**,** with pathway significance assessed using FDR-adjusted *p*-values and pathway impact scores determined based on metabolite topological analysis within each pathway.

#### 4.4.5. Metabolomics Outlier Identification and Quality Control

The LC-MS/MS analysis results were analyzed using an in-house Python (version 3.10; Python Software Foundation, https://www.python.org/) script to identify outliers and validate data quality and integrity based on Euclidean distance calculations instead of being limited to a visual PCA exploration. Metabolites with more than 80% missing values were excluded from the study. Data processing and formatting were performed with Pandas (version 2.0.3; pandas development team, https://pandas.pydata.org/). A log transformation was applied using NumPy (version 1.25.2; NumPy Developers, https://numpy.org/) to address skewness. The data were standardized utilizing Scikit-learn’s StandardScaler (version 1.3.0; scikit-learn developers, https://scikit-learn.org/), after which pairwise Euclidean distance calculations were performed. A heatmap and hierarchical clustering were developed using Seaborn (version 0.12.2; Seaborn Developers, https://seaborn.pydata.org/) and Plotly (version 5.15.0; Plotly Technologies Inc., Montréal, QC, Canada, https://plotly.com/) to visualize the distance matrix based on the calculated distances to detect outliers.

### 4.5. Kinetic Analysis of Planktonic Bacteria and pH Measurements

Bacterial suspensions of OD_600nm_ of 0.1 in TSBG were treated with different concentrations of AEA (12.5–25 μg/mL) and incubated at 37 °C, in air/5% CO_2_, for 24 h. At various time points, the pH of the samples was measured using pH indicator paper strips (MColorpHast, Merck KGaA, Darmstadt, Germany), and the OD_600nm_ was measured in parallel in a Tecan Infinite M200 PRO plate reader (Tecan Trading AG, Männedorf, Switzerland) [12]. Untreated samples served as a control.

### 4.6. Acid Tolerance Assay

Overnight bacterial suspensions of OD_600nm_ of 0.1 in TSBG were centrifuged and resuspended in either TSBG pH 7, or in TSBG with the pH adjusted to 5.2 [36,37] by the addition of 0.2 M disodium hydrogen orthophosphate and 0.1 M citric acid (each at a final concentration of approximately 20 mM). Samples were treated (or untreated for control) with AEA (10 µg/mL) and incubated at 37 °C, air/5% CO_2_, for 0, 1, and 6 h. Growth characteristics were determined by culturing *S. mutans* in 200 µL aliquots of the medium in a 96-well sterile microtiter tray. Bacterial growth was measured using the OD_600nm_ in a Tecan Infinite M200 PRO plate reader (Tecan Trading AG).

In parallel, to assess bacterial adaptation to acid tolerance, samples were taken before each time point and serially diluted from 10^−1^ to 10^−6^. Then, 10 µL from each suspension was plated on BHI (Acumedia, Lansing, MI, USA) agar plates and incubated at 37 °C, air/5% CO_2_, overnight. The CFUs were counted, and the results are expressed as the mean percentage (±standard deviation) of colonies present [36,37].

The following equation was used to calculate the CFUs per well in the original sample: number of colonies × dilution factor × original volume of sample × 10 [12].

### 4.7. Carbohydrate Rescue Assay

Overnight bacterial suspensions in TSBG were centrifuged and resuspended in TSB to an OD_600nm_ of 0.1 with the addition of carbohydrates such as fructose, mannitol, galactose, and lactose (glucose served as a control). Samples were treated with AEA (1.25–50 µg/mL) and incubated at 37 °C, in air/5% CO_2_, overnight. The absorbance of overnight bacterial growth was recorded at OD_600nm_ in the Tecan Infinite M200 PRO plate reader (Tecan Trading AG) [12].

### 4.8. Statistical Analysis

All experiments, with the exception of metabolomics experiments, were independently performed three times in triplicate. Data were statistically analyzed using Student’s *t*-test in Microsoft Excel, with a *p*-value of less than 0.05 considered significant when comparing treated versus control samples.

## Data Availability

The original mass spectrometry metabolomics data in this study are openly available at the MassIVE repository at ftp://massive-ftp.ucsd.edu/v09/MSV000097584/ (accessed on 27 August 2025) with the data set identifierMSV000097584.

## Supplementary Materials

The following supporting information can be downloaded at https://www.mdpi.com/article/10.3390/ijms26178401/s1.

## Abbreviations

The following abbreviations are used in this manuscript:

*S. mutans*	*Streptococcus mutans*
AEA	Anandamide
MIC	Minimum inhibitory concentration
ATP	Adenosine triphosphate
LC-MS	Liquid chromatography–mass spectrometry
2-AG	2-arachidonoylglycerol
CB1/CB2	Cannabinoid receptor 1/2
NAPE	*N*-acyl-phosphatidylethanolamie
PIP2	Phosphatidylinositol-4,5-bisphosphate
GPCRs	G-protein-coupled receptors
MRSA	Methicillin-resistant *Staphylococcus aureus*
TSB	Tryptic soy broth
TSBG	Tryptic soy broth with 1% glucose
OD	Optical density
DDW	Double-distilled water
PBS	Phosphate-buffered saline
HPLC	High-performance liquid chromatography
QC	Quality control
UPLC	Ultimate 300 high-performance liquid chromatography
m/z	Mass/charge ratio
PCA	Principal component analysis
BHI	Brain heart infusion
CFUs	Colony-forming units
G6P	Glucose-6-phosphate
F6P	Fructose-6-phosphate
F1,6BP	Fructose-1,6-bisphosphate
2-PG	2-phosphoglycerate
PEP	Phosphoenolpyruvate
GA3P	Glyceraldehyde-3-phosphate
PPP	Pentose phosphate pathway
EPS	Extracellular polysaccharides
X5P	Xylitol-5-phosphate
PTS	Phosphotransferase system
EI	Enzyme I
EII	Enzyme II
HPr	Heat-stable phosphocarrier protein
ABCs	ATP-binding cassettes
PDH	Pyruvate dehydrogenase
