# Supplementary material for: Anandamide Alters Glycolytic Activity in Streptococcus mutans: Metabolomics and Stable Isotope Labeling Study
Source: Int J Mol Sci. 2025 Aug 29;26(17):8401. doi: 10.3390/ijms26178401 (PMC12429060; doi:10.3390/ijms26178401)
Supplement: Supplementary file 1 [file ijms-26-08401-s001.zip › Figure S1.pdf]

Figure S1: AEA Treatment Causes a delay and reducton in the Growth of S. mutans

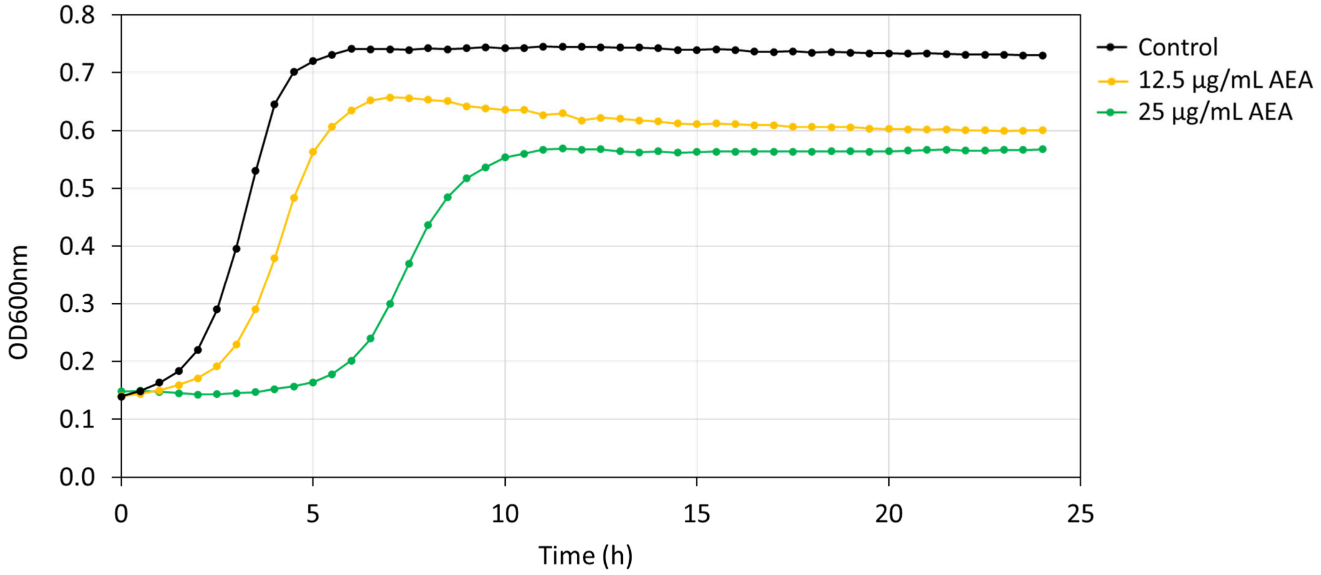

Figure S1. AEA causes a delay in the growth curve of *S. mutans.* (**a**) The OD of *S. mutans* measured over the span of a 24 h incubation with AEA (0, 12.5, and 25µg/mL). *n* = 3.
